# GIANT: pattern analysis of molecular interactions in 3D structures of protein–small ligand complexes

**DOI:** 10.1186/1471-2105-15-12

**Published:** 2014-01-14

**Authors:** Kota Kasahara, Kengo Kinoshita

**Affiliations:** 1 Graduate School of Information Sciences, Tohoku University, 6-3-09 Aoba, Aramaki, Aoba-ku, Sendai, Miyagi 980-8597, Japan; 2 Tohoku Medical Megabank Organization, Tohoku University, 2-1 Seiryo-cho, Aoba-ku, Sendai, Miyagi 980-8573, Japan; 3 Institute of Development, Aging, and Cancer, Tohoku University, 4-1 Seiryo-cho, Aoba-ku, Sendai, Miyagi 980-8575, Japan; 4 Present addresses: Institute for Protein Research, Osaka University, 3-2 Yamada-oka, Suita, Osaka 565-0871, Japan

**Keywords:** Molecular recognition, Ligand binding site, Protein–ligand interactions, Protein structure, Protein function, Pattern recognition, Database, Web-server

## Abstract

**Background:**

Interpretation of binding modes of protein–small ligand complexes from 3D structure data is essential for understanding selective ligand recognition by proteins. It is often performed by visual inspection and sometimes largely depends on *a priori* knowledge about typical interactions such as hydrogen bonds and π-π stacking. Because it can introduce some biases due to scientists’ subjective perspectives, more objective viewpoints considering a wide range of interactions are required.

**Description:**

In this paper, we present a web server for analyzing protein–small ligand interactions on the basis of patterns of atomic contacts, or “interaction patterns” obtained from the statistical analyses of 3D structures of protein–ligand complexes in our previous study. This server can guide visual inspection by providing information about interaction patterns for each atomic contact in 3D structures. Users can visually investigate what atomic contacts in user-specified 3D structures of protein–small ligand complexes are statistically overrepresented. This server consists of two main components: “Complex Analyzer”, and “Pattern Viewer”. The former provides a 3D structure viewer with annotations of interacting amino acid residues, ligand atoms, and interacting pairs of these. In the annotations of interacting pairs, assignment to an interaction pattern of each contact and statistical preferences of the patterns are presented. The “Pattern Viewer” provides details of each interaction pattern. Users can see visual representations of probability density functions of interactions, and a list of protein–ligand complexes showing similar interactions.

**Conclusions:**

Users can interactively analyze protein–small ligand binding modes with statistically determined interaction patterns rather than relying on *a priori* knowledge of the users, by using our new web server named GIANT that is freely available at http://giant.hgc.jp/.

## Background

Elucidating molecular mechanisms in the selective recognition of small molecules (or ligands) by proteins is a central issue in biology. Structure data of protein–ligand complexes deposited in Protein databank (PDB) [1] are a very informative resource because the data contain direct information of molecular interactions between proteins and ligands at the atomistic scale. Structural biologists and medicinal chemists can obtain implications and knowledge through visual inspection of 3D structures of protein–ligand complexes. However, visual inspections by scientists are subjective and may focus on only some particular well-known interactions, e.g. hydrogen bonds. A more objective and comprehensive view is required for the interpretation of molecular interactions from 3D structure data.

Toward more objective analyses, a promising strategy is taking advantages of statistics of molecular interactions on PDB. Due to recent rapid increase in 3D structure data, this strategy has been become more attractive, and the statistics of protein–ligand interactions have been extensively studied [2]. Many secondary databases of PDB focusing on protein–ligand complexes with various annotations have been constructed [3-7]. Relibase [8], CREDO [9] and PLI [10] particularly focus on atomic contacts between proteins and ligands. They store information of protein–ligand interactions at the atomistic level and provide catalogues of many types of interactions, such as hydrogen bonds, hydrophobic contacts and interactions of π-systems. While they provide fruitful information about protein–ligand interactions, analyses with these existing databases are limited to well-known, preliminarily defined atomic contacts. However, it is considered that the selective molecular recognition is accomplished by combinations of not only such typical interactions but also a huge variety of atomic contacts. Comprehensive knowledge about various kinds of atomic contacts is required.

Previously, we reported a comprehensive classification of spatial arrangements of ligand atoms around molecular fragments of proteins that were defined as three covalently linked atoms [11]. We analyzed statistically preferred geometries of the atomic contacts, or interaction patterns, as mixtures of Gaussian functions. These interaction patterns were obtained from every atomic contact observed in PDB using an unsupervised pattern recognition approach [12]. We found 13,512 interaction patterns in PDB and interactions in these patterns were more enriched in native complex structures than in docking decoys.

On the basis of the classification of interactions, we present a new web server for analyzing molecular interactions in the 3D structure of protein–ligand complexes, named “GIANT”, which stands for “Gaussian mixture model-based Interaction ANalyzer focusing on Three-atom fragments”. GIANT provides a web browser-based user interface for visual inspection of protein–ligand interactions in 3D structures. Users can investigate how statistically overrepresented each atomic contacts in the PDB, and what protein–ligand complex uses similar interactions, for any kind of atomic contacts rather than well-known predefined types of interactions.

## Construction and content

**Figure 1:**
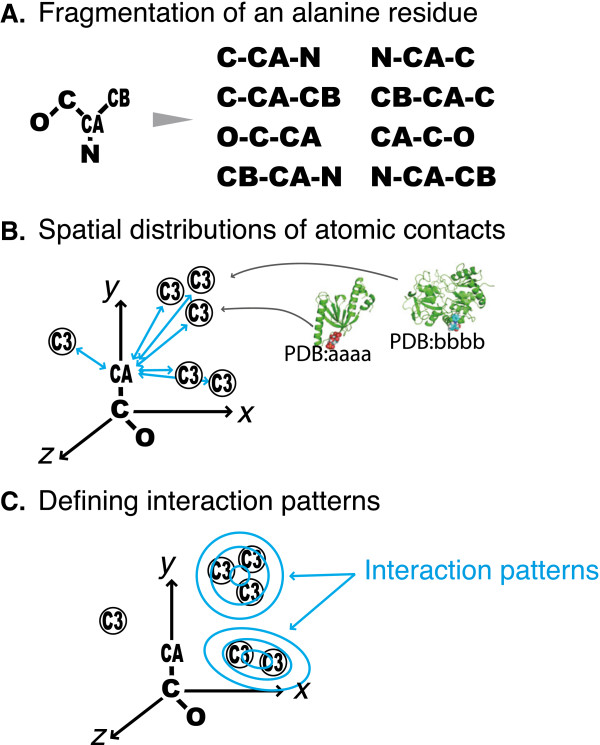
**Methods for defining interaction patterns. (A)** As an example of fragmentation of amino acid residues, the case of alanine is illustrated, where eight fragments were obtained: N-CA-CB, C-CA-CB, C-CA-N, O–C-CA, and the fragments with the reverse orders. **(B)** Contacting pairs of a protein fragment and a ligand atom were collected from the dataset. When the distance between the first atom of the fragment and the ligand atom was less than the criterion (the sum of van der Waals radii plus the offset value 1.0 Å), the pair was sampled. Sampled contacting pairs formed a 3D spatial distribution on the basis of reference coordinates defined by the three atoms in the protein fragment. Three arrows indicate the axes of the reference coordinate system, “CA-C-O” is a protein fragment, and “C3” in circles denote positions of interacting ligand atoms observed in the dataset (“C3” means a type of ligand atom i.e., a sp3 carbon atom). **(C)** A pattern recognition technique was applied to each 3D spatial distribution. Interaction patterns were defined as Gaussian mixture distributions.

As in the previous paper, the dataset consists of 3D structures of 66,654 protein–ligand binding sites from 23,040 PDB entries. These entries were chosen from a snapshot of PDB on Sept. 25^th^, 2010 using following criteria: (1) structures had been determined by X-ray crystallography with resolution ≤2.5 Å, (2) receptor proteins had ≥30 amino acid residues, and (3) bound ligands have the molecular weight between 80 and 800 Da, contained >5 atoms, and had < 0.6 relative accessible surface area. For each protein in the dataset, amino acids residues were decomposed into fragments consisting of three covalently linked atoms (Figure 1A). Ligand atoms were classified on the basis of Tripos force field [13]. In the dataset, there were 565 types of protein fragments and 27 types of ligand atoms. From the complex structures, interacting pairs of protein fragments and ligands atom were collected, and the spatial distributions of interacting ligand atoms relative to the protein fragments were considered (Figure 1B). Then, a pattern recognition technique was applied to infer parameters of Gaussian mixture models with the best fits to the spatial distributions, and 13,519 interaction patterns (i.e., Gaussian functions) were found (Figure 1C) for 8,022 types of combinations of the protein fragment and ligand atom. When the Mahalanobis distance between an interaction pattern and a position of atomic contacts, was ≤2.5, the interaction pattern was annotated onto the contacting pair. 63.4% of ligand atoms in our non-redundant dataset were recognized by at least one interaction pattern; for each complex, more than half of ligand atoms recognized with at least one interaction pattern for the cases of 70.5% of complexes. See our previous paper for details about the statistics and the methods [11].

On the basis of the analyses, we constructed a web-based application called GIANT, which consists of two main components: “Complex Analyzer” and “Pattern Viewer”. The former provides functionality to perform visual inspection of protein–ligand interactions on the basis of annotations of interaction patterns, and the latter shows a summary of each interaction pattern.

## Utility and discussion

We show two examples of analyses of protein–ligand interactions. In the first example, a brief analysis of binding modes of the dihydrofolate reductase (DHFR) and methotrexate (MTX) complex [PDB:3dfr] [14] was described in a tutorial-style whitch instructs basic usage of GIANT. The second one is a practical case that compares interactions of two similar inhibitors recognized with the same binding mode by identical cycline-dependent kinase (CDK) [PDB:2r3j, 2r3k] [15].

### Tutorial with a DHFR–methotrexate complex

We show a tutorial-style example for analyzing interactions of a DHFR–methotrexate complex. In this example, it is assumed that users want to know the molecular mechanisms of methotrexate recognition by DHFR. We here aim to study what kinds of statistically preferred interactions are working on the recognition of the pteridine ring and what other complexes apply similar interactions, by using GIANT.

**Figure 2:**
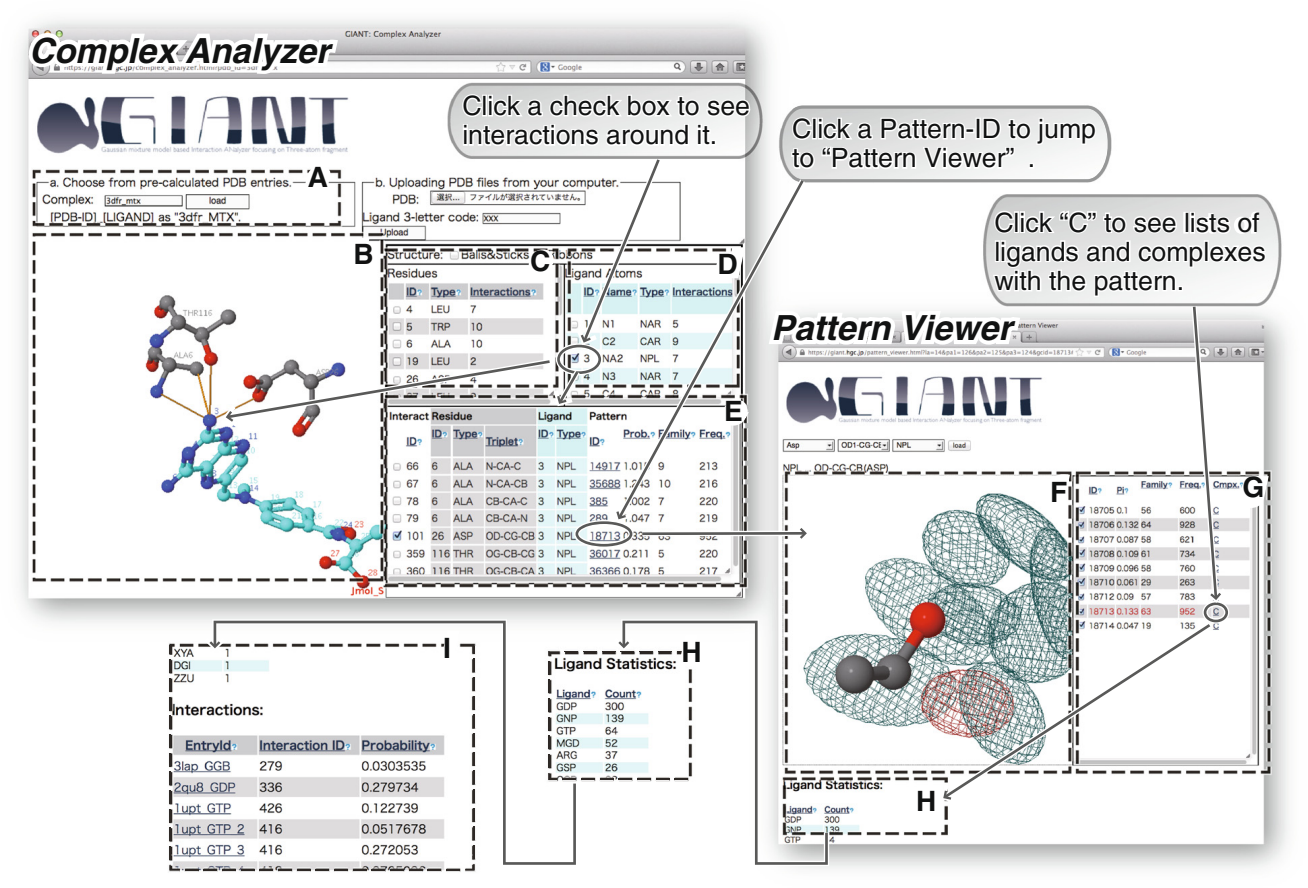
**Screenshots of GIANT consisting of the two parts: complex analyzer (A-E) and pattern viewer (F-I). (A)** Query input box. **(B)** 3D structure viewer for a query protein–ligand complex. **(C)** List of interacting residues. **(D)** List of interacting ligand atoms. **(E)** List of interactions. **(F)** 3D viewer for spatial distribution of interaction patterns. The red contour is the interaction pattern that is specified in Complex Analyzer by users. **(G)** List of interaction patterns. The row shown in red corresponds to the red contour in the 3D viewer. **(H)** List of ligands recognized with a user-specified interaction pattern. **(I)** List of protein–ligand complexes using a user-specified interaction pattern.

Users should first specify a query complex by designating a PDB-ID with a ligand 3-letter code or by uploading a flat file in PDB format with specifying ligand 3-letter code. Our example employs the former method. In this flow, users should open the page “Complex Analyzer” at the top page and input a PDB-ID with a ligand 3-letter code such “3dfr_MTX” (Figure 2A). Following this, users can observe the complex structure in the Jmol (http://jmol.org/) applet (Figure 2B) by clicking the ‘load’ button (Figure 2A).

On the right side of the window, there are three tables: a list of interacting amino acid residues (Figure 2C), interacting ligand atoms (Figure 2D) and interacting pairs of a protein fragment and a ligand atom (Figure 2E). Users can view interactions in the Jmol viewer by clicking check boxes in the tables. For example, they can focus on interactions of the 2′ amino group in the pteridine ring in MTX by filling the corresponding check box (Atom-ID = 3) in the table of ligand atoms. Amino acid residues recognizing the specified atom will then appear, and interactions will be depicted as lines between atoms (Figure 2B). Furthermore, the table of interaction collectively shows a list of interactions of the specified atom. In this case, the nitrogen atom composing the amino group is recognized by three residues: Ala6, Asp26 and Thr116. The interaction with Asp26 is a bifurcated hydrogen bond. This interaction pattern (Pattern-ID = 18713) was widely observed in the dataset, i.e., 952 interactions in 63 protein families (defined by a single-linkage cluster of amino acid sequences within 25% sequence identity with ≥50% sequence coverage) were assigned to this pattern. These values are described in the column “Freq.” and “Family” in the table of interactions. Users can also see the competence of this interaction to a probability distribution by checking the value in the column “Prob.” that denotes the probability density of this data point in the Gaussian mixture distribution. In contrast to that the interaction pattern of Asp26 with the nitrogen atom that is a common feature in a wide range of protein families, the interaction with Thr116 was observed only in five protein families. This interaction pattern is almost specific to the DHFR family (This residue recognizes the ligand via an intermediate water molecule. Although GIANT does not have information about water molecules, it shows such interactions as direct contacts provided distances between contacting atoms are below a threshold, calculated as sum of van der Waals radii and 1.0 Å).

While “Complex Analyzer” provides information about assignments of atomic contacts to interaction patterns for user-specified complexes, it is still difficult to interpret the nature of each interaction pattern using only this component. The alternative component “Pattern Viewer” helps analyses by providing graphical information about the 3D spatial probability distribution of each interaction pattern. Users can jump to this component by clicking “Pattern-ID” in any row of the table of interactions. To see the interaction patterns with Asp26, click “18713” (the seventh column) of the row with Interaction-ID = 101 (the first column) in the table of interactions. The “Pattern Viewer” page will be opened and will show the spatial distribution of each interaction pattern as 3D meshes (Figure 2F). Three covalently linked atoms centered in the viewer represent a fragment of proteins. The regions inside of the meshes are statistically preferred positions of ligand atoms for interaction with the fragment. The contour of the pattern specified in the “Complex Analyzer” is highlighted in red. Clicking “C” in the right table (Figure 2G) provides a list of ligands (Figure 2H) and that of protein–ligand complexes (Figure 2I) with the same interaction patterns. This information may be useful for seeking complexes with similar interactions to the query.

### Comparing interactions of two CDK inhibitors

**Figure 3:**
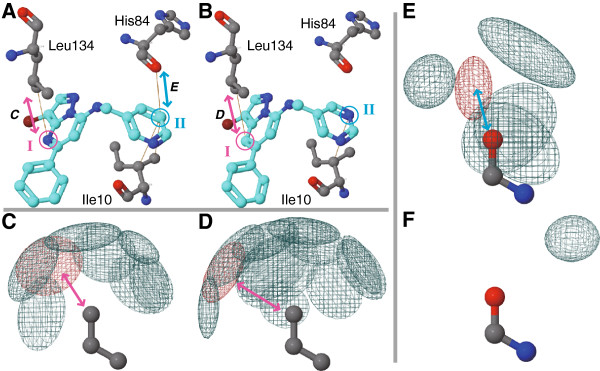
**Differences in interactions between two similar compounds bound with an identical target protein, CDK. (A, B)** Screenshots of Complex Analyzer for [PDB: 2r3j] and [PDB: 2r3k]. Circles I and II indicate altered atoms between these two ligands. Three amino acid residues interacted with these two altered atoms, i.e., Ile10, His84, and Leu134, were shown as a stick model. Pink and Cyan arrows emphasizes interactions in patterns, and they corresponds to the panel **(C, D)** and **(E)**, respectively. **(C, D, E, F)** Screenshots of Pattern Viewer about the interactions of Leu Cδ-Cγ-Cβ fragments with aromatic nitrogen atoms **(C)** and with aromatic carbon atoms **(D)**, and those of His O-C-N with aromatic carbon atoms **(E)** and aromatic nitrogen atoms **(F)**.

Subtle structural differences in a compound can drastically change its binding affinity to a target protein without disruption of hydrogen bonds and such well-known interactions. Since the complexity in structure–activity relationships is a major barrier in drug discovery projects, understanding preferences of interactions in each atom is important. GIANT provides some clues to that from the statistical perspective. As an example, we show analyses on differences between two similar ligands binding with an identical target protein, CDK. The structures of these two ligands were shown in Figure 3A [PDB: 2r3j] and B [PDB: 2r3k] (they were not in the pre-calculated dataset thus users need upload the PDB files). Although the differences in chemical structures between these ligands were only two atoms, that marked as I and II in Figure 3A and B, IC50 value of the ligand in 2r3j was 10-fold lower than that in 2r3k [15]. What makes this significant difference in binding affinity is unclear, because these atoms did not make hydrogen bonds with the binding sites and there was no significant structural change in amino acid residues in binding sites.

While one of the two altered parts in the ligands had similar interactions between 2r3j and 2r3k, the other one had distinct interactions. In the position I, that was an aromatic nitrogen atom and aromatic carbon atom in 2r3j and 2r3k, respectively, interacted with Leu134 residue by a CH–π interaction. The spatial distributions of aromatic nitrogen and carbon atoms interacting with Leu Cδ–Cγ–Cβ fragment were similar (Figure 3C and D, respectively), and both of patterns used in these atoms (shown as red contours) were widely shared in many protein families (89 and 174 families shares the patterns for aromatic nitrogen and aromatic carbon atoms, respectively). On the other hand, the position II, that is an aromatic carbon atom in 2r3j and an aromatic nitrogen atom in 2r3k, contacted with Ile10 side-chain and His84 main-chain. While interactions between Ile10 and the position II was in a pattern for the both complexes, that between His84 and the position II were in a pattern only for the complex 2r3k (position II was an aromatic carbon atom) despite of there was no significant structural changes (interatomic distance between the His84 backbone oxygen atom and the contacting ligand atoms were 3.7 Å and 3.6 Å in 2r3j and 2r3k, respectively). In contrast to the spatial distribution of aromatic carbon atoms around His O–C-N fragment (Figure 3E), that of aromatic nitrogen atoms preferred only one configuration of interactions (Figure 3F). This result implies that this loss of the statistically preferred interactions with His84 main chain causes 10-fold gain of IC50 value in 2r3k complex from 2r3j, and the position II should be a carbon atom rather than a nitrogen atom for higher affinity. This should be a helpful information for medicinal chemists.

### Future perspective

Although the scope of GIANT is limited to the direct contacts between proteins and small molecules in the current version, the basic concept of GIANT is applicable to other various kinds of molecular interactions such as water-mediated interactions. In the future developments, we are planning taking statistics of interactions with metal and water molecules that play important roles for molecular recognitions. In addition, while the interaction patterns defined in GIANT focuses on the relative positions between a protein fragment and a ligand atom, and does not consider the combination of the elements interactions (or the “environment” around the contacting pair). The information about environment should be an important factor in the ligand recognition, we will take some statistics of co-occurrences of the interaction patterns in a future work.

## Conclusions

The web-server GIANT shows the statistical preferences of each atomic contact in user specified 3D structures of protein–small ligand complexes. This function provides an objective perspective for visual inspections of binding modes on the basis of results of the survey of interactions reported in the previous paper, and provides many implications for structural biologists and medicinal chemists. For example, when medicinal chemists perform lead optimization with 3D structure data of protein–compound complexes, GIANT suggests parts of compounds where chemical moieties without statistically overrepresented interaction patterns should be replaced to gain binding affinities. Although this process has usually been performed by experts using their *a priori* knowledge and intuition, GIANT supports it with statistical, objective information and assists in realizing the concept of so-called rational drug-design.

## Availability and requirement

GIANT is freely available at the following URL: http://giant.hgc.jp/.

## Abbreviations

DHFR: Dihydrofolate reductase; MTX: Methotrexate; CDK: Cyclin-dependent kinase.

## Competing interests

The authors declare that they have no competing interests.

## Authors’ contributions

K Kasahara performed researches and implemented the web-server. K Kasahara and K Kinoshita designed this work, and wrote the manuscript. Both authors read and approved the final manuscript.
